# Mental health of people in Australia in the first month of COVID‐19 restrictions: a national survey

**DOI:** 10.5694/mja2.50831

**Published:** 2020-10-26

**Authors:** Jane RW Fisher, Thach D Tran, Karin Hammarberg, Jayagowri Sastry, Hau Nguyen, Heather Rowe, Sally Popplestone, Ruby Stocker, Claire Stubber, Maggie Kirkman

**Affiliations:** ^1^ Monash University Melbourne VIC; ^2^ Victorian Assisted Reproductive Treatment Authority Melbourne VIC

**Keywords:** COVID‐19, Mental health policy, Depressive disorders, Anxiety disorders, Suicide, Infectious diseases, Respiratory tract infections

## Abstract

**Objectives:**

To estimate the population prevalence of clinically significant symptoms of depression, generalised anxiety, thoughts of being better off dead, irritability, and high optimism about the future, and of direct experience of COVID‐19, loss of employment caused by COVID‐19 restrictions, worry about contracting COVID‐19, or major disadvantage because of the restrictions; to examine the relationship between these experiences and reporting mental symptoms.

**Design, setting, participants:**

Anonymous online survey of adult Australian residents, 3 April – 2 May 2020.

**Main outcome measures:**

Self‐reported psychological status during the preceding fortnight assessed with the Patient Health Questionnaire 9 (PHQ‐9; symptoms of depression) and the Generalised Anxiety Disorder Scale (GAD‐7). Optimism about the future was assessed with a 10‐point study‐specific visual analogue scale.

**Results:**

13 829 respondents contributed complete response data. The estimated prevalence of clinically significant symptoms of depression (PHQ‐9 ≥ 10) was 27.6% (95% CI, 26.1–29.1%) and of clinically significant symptoms of anxiety (GAD‐7 ≥ 10) 21.0% (95% CI, 19.6–22.4%); 14.6% of respondents (95% CI, 13.5–16.0%) reported thoughts of being better off dead or self‐harm (PHQ‐9, item 9) on at least some days and 59.2% (95% CI, 57.6–60.7%) that they were more irritable (GAD‐7, item 6). An estimated 28.3% of respondents (95% CI, 27.1–29.6%) reported great optimism about the future (score ≥ 8). People who had lost jobs, were worried about contracting COVID‐19, or for whom the restrictions had a highly adverse impact on daily life were more likely to report symptoms of depression or anxiety, and less likely to report high optimism than people without these experiences.

**Conclusions:**

Mental health problems were widespread among Australians during the first month of the stage two COVID‐19 restrictions; in addition, about one‐quarter of respondents reported mild to moderate symptoms of depression or anxiety. A public mental health response that includes universal, selective and indicated clinical interventions is needed.

**The known**: No Australian national population data about mental health related to COVID‐19 restrictions have been published.

**The new**: During the first month of COVID‐19 restrictions, the prevalence of clinically significant symptoms of depression and generalised anxiety, thoughts of being better off dead, and irritability were markedly higher than reported by previous surveys, particularly among people who had lost jobs because of the restrictions, were worried about contracting COVID‐19, or for whom the restrictions had a major impact on their daily lives.

**The implications**: A public mental health response encompassing universal, selective, and indicated strategies is needed in the health and other sectors.

Public health measures to limit the spread of coronavirus disease 2019 (COVID‐19) include requirements not to leave home except for specified purposes, to work from home when practical, to limit proximity to other people, to not visit residential aged care homes, to limit the number of people at social events (weddings, funerals, celebrations), to restrict interstate and international travel, and to accept the enforcement of these restrictions. The mental health consequences of these measures are likely to be unevenly distributed across the community because they also depend on individual social and economic circumstances.

A recent position paper^1^ summarised international expert opinion on research priorities for mental health during the COVID‐19 pandemic. The first recommendation was to gather high quality population level data on its mental health effects. The aim of our study was to assess the mental health of people in Australia during the first month of COVID‐19‐related restrictions. Our specific objectives were to estimate population prevalence rates of clinically significant symptoms of depression, generalised anxiety, thoughts of being better off dead, increased irritability, and high optimism about the future; to estimate the prevalence of direct experiences of COVID‐19, loss of employment caused by COVID‐19 restrictions, concern about contracting COVID‐19, and major disadvantage because of the restrictions; and to assess associations between these experiences and mental health symptoms.

## Methods

A short, anonymous survey (estimated completion time, 8 minutes) of people living in Australia and aged at least 18 years was available on the Monash University website (https://www.monash.edu/medic​ine/living-with-covid-19-restr​ictio​ns-survey) from 3 April 2020 (four days after national stage two COVID‐19 restrictions were announced by the Prime Minister; phase one restrictions had been gradually introduced during March) until midnight on 2/3 May 2020 (further information: online Supporting Information).

### Mental health

Psychological symptoms experienced during the preceding fortnight were assessed with the Patient Health Questionnaire 9 (PHQ‐9) and the Generalised Anxiety Disorder Scale (GAD‐7). The PHQ‐9^2^ is an easily understood scale that asks respondents to rate their experience of nine symptoms from 0 (not experienced) to 3 (experienced nearly every day); a total PHQ‐9 score of 10 or more indicates clinically significant (moderate to severe) symptoms, while scores of 5–9 indicate mild symptoms. The GAD‐7^3^ is an easily understood scale that asks respondents to rate their experience of seven symptoms of anxiety with the same response options as the PHQ‐9; a total GAD‐7 score of 10 or more indicate clinically significant (moderate to severe) symptoms, while scores of 5–9 indicate mild symptoms.

Optimism about the future was assessed with a visual analogue scale (from 0, not at all optimistic, to 10, extremely optimistic). We defined scores of 8 or more as indicating great optimism, and scores of 0–7 as indicating no to moderate optimism about the future.

### Experience of COVID‐19 and COVID‐19 restrictions

We asked whether respondents had direct experience of COVID‐19 (diagnosis with or testing for COVID‐19, or living with or knew someone with COVID‐19), or had lost employment because of COVID‐19 restrictions; how worried they were about contracting COVID‐19 (visual analogue scale, 0–10); and to what degree COVID‐19 restrictions had affected daily life (visual analogue scale, 0–10).

### Socio‐demographic characteristics

We asked questions with fixed response options to ascertain age, sex, residential postcode, birthplace (Australia or overseas), living circumstances, and occupation. Information about state of residence, remoteness (urban or rural), and socio‐economic position (Index of Relative Socio‐economic Advantage and Disadvantage) were derived from respondents’ postcodes using the most recent Australian Bureau of Statistics data.^4^ Further details about the survey are included in the online Supporting Information.

### Statistical analysis

Population prevalence rates with 95% confidence intervals (CIs) were estimated for mental health assessment parameters and experiences of COVID‐19 and restrictions, adjusted for differences in selected socio‐demographic characteristics (state, socio‐economic position decile, sex, age) between the respondents and the Australian population at September 2019.^4^

The characteristics of respondents with experiences of COVID‐19 and related restrictions, and associations between mental health assessment parameters and experiences of COVID‐19 and restrictions were assessed by multiple logistic regression, adjusted for selected socio‐demographic characteristics.

All analyses were undertaken in Stata 16. Further details about the statistical analysis are included in the Supporting Information.

### Ethics approval

Our investigation was approved by the Monash University Human Research Ethics Committee (reference, 2020‐24080‐42716).

## Results

After excluding 80 respondents not resident in Australia and 33 under 18 years of age, there were 15 121 eligible respondents; 13 829 (91.5%) contributed complete data and were included in our analyses. Respondents were drawn from all Australian states and territories, socio‐economic positions, age groups, and living situations. Compared with the national population, similar proportions were born overseas, the proportion from Victoria was larger and that from New South Wales smaller, the proportion of women was higher, and the socio‐economic position distribution was skewed to higher levels (Box 1).

Box 1 **Socio‐demographic characteristics of the 13 829 eligible survey respondents who provided complete response data**

**Table:** 

	Respondents	All Australian adults^5^
State/territory		
New South Wales	2753 (19.9%)	32.1%
Victoria	6105 (44.1%)	26.2%
Queensland	1939 (14.0%)	19.8%
Western Australia	1177 (8.5%)	10.2%
South Australia	836 (6.0%)	7.0%
Tasmania	445 (3.2%)	2.1%
Australian Capital Territory	465 (3.4%)	1.7%
Northern Territory	109 (0.8%)	0.9%
Socio‐economic position*		
Quintile 1 (lowest)	1093 (7.9%)	16.8%
Quintile 2	1541 (11.1%)	17.2%
Quintile 3	2228 (16.1%)	20.7%
Quintile 4	3038 (22.0%)	20.5%
Quintile 5 (highest)	5929 (42.9%)	24.8%
Sex		
Women	10 434 (75.5%)	50.9%
Men	3328 (24.1%)	49.1%
Other	67 (0.5%)	NA
Age (years)		
18–29	1337 (9.7%)	21.8%
30–39	2294 (16.6%)	18.6%
40–49	2854 (20.6%)	16.6%
50–59	3064 (22.2%)	15.6%
60–69	2833 (20.5%)	13.2%
70 or more	1447 (10.5%)	14.2%
Living situation		
On your own	2660 (19.2%)	NA
With partner/partner and children/adult family members	9630 (69.6%)	NA
With children and without a partner	578 (4.2%)	NA
In a shared house with non‐family members/other	961 (6.9%)	NA
Place of birth		
Australia	10 679 (77.2%)	70.3%
Overseas	3150 (22.8%)	29.7%
Main occupation (before COVID‐19)		
Paid job	8330 (60.2%)	NA
Unpaid work caring for children/dependent relatives only, or unemployed	1146 (8.3%)	NA
Student	1343 (9.7%)	NA
Retired	3010 (21.8%)	NA

NA = not available.

* Index of Relative Socio‐economic Advantage and Disadvantage.

### Experience of COVID‐19 and related restrictions

Eighteen respondents had contracted COVID‐19 and been admitted to hospital (weighted proportion, 0.18%), 38 had contracted COVID‐19 but not been admitted to hospital (0.26%), 539 had been tested (4.1%), 47 lived with someone who had been COVID‐19‐positive (0.49%), and 1699 knew but did not live with someone who had been COVID‐19‐positive (11.8%). The estimated proportion of respondents with any direct experience of COVID‐19 was 15.3%; 11.2% had lost their jobs because of COVID‐19, 13.9% were very worried about contracting COVID‐19, and 25.2% reported a major negative impact of the restrictions (Box 2).

Box 2 **Experiences of COVID‐19 and related restrictions among 13 829 survey respondents**

**Table:** 

Experience	Number	Estimated prevalence* (95% CI)
Diagnosed with or tested for COVID‐19, or knew someone diagnosed with COVID‐19	2147	15.3% (14.2–16.4%)
Diagnosed with COVID‐19, admitted to hospital	18	0.18% (0.09–0.38%)
Diagnosed with COVID‐19, not admitted to hospital	38	0.26% (0.14–0.46%)
Tested for COVID‐19	539	4.1% (3.6–4.7%)
Lived with someone diagnosed with COVID‐19	47	0.49% (0.31–0.77%)
Knew someone diagnosed with COVID‐19	1699	11.8% (10.8–12.8%)
Lost a job because of COVID‐19 restrictions	1251	11.2% (10.0–12.4%)
Highly worried about contracting COVID‐19 (scale score ≥ 8)	2185	13.9% (13.1–14.8%)
High impact of restrictions (scale score ≥ 8)	3435	25.2% (23.8–26.8%)

CI = confidence interval.

* Weighted by state, socio‐economic position decile, sex, and age.

Respondents living in Victoria, Queensland, Western Australia and the Australian Capital Territory were less likely to have had direct experience of COVID‐19 than those from NSW. Respondents in areas of highest socio‐economic position or born overseas were more likely to report direct experience of COVID‐19; people who were at least 70 years old, retired, or caring for dependent relatives at home were less likely to have had direct experience of COVID‐19 (Box 3).

Box 3 Characteristics of respondents with direct experiences of COVID‐19: adjusted odds ratios* with 95% confidence intervals

**Table:** 

	Any direct experience of COVID‐19	Lost a job because of COVID‐19	Greatly worried about contracting COVID‐19	High negative impact of COVID‐19 restrictions
State				
New South Wales	1	1	1	1
Victoria	0.79 (0.70–0.89)	1.03 (0.87–1.21)	1.01 (0.90–1.15)	1.14 (1.02–1.26)
Queensland	0.85 (0.72–0.99)	1.20 (0.97–1.48)	0.95 (0.81–1.11)	1.07 (0.93–1.23)
Western Australia	0.61 (0.49–0.74)	1.10 (0.86–1.40)	0.78 (0.64–0.95)	0.83 (0.70–0.98)
South Australia	0.96 (0.77–1.19)	0.85 (0.63–1.16)	0.89 (0.72–1.10)	0.84 (0.69–1.01)
Tasmania	0.93 (0.69–1.25)	0.94 (0.63–1.39)	0.74 (0.55–1.00)	1.00 (0.78–1.29)
Australian Capital Territory	0.62 (0.46–0.82)	0.47 (0.29–0.76)	0.74 (0.55–1.00)	0.72 (0.56–0.93)
Northern Territory	0.64 (0.36–1.12)	0.51 (0.23–1.14)	0.63 (0.33–1.20)	0.61 (0.35–1.05)
Major city (*v* regional/remote areas)	0.98 (0.86–1.12)	0.82 (0.69–0.96)	1.05 (0.92–1.18)	1.28 (1.15–1.43)
Socio‐economic status†				
Quintile 1 (lowest)	1	1	1	1
Quintile 2	1.06 (0.83–1.35)	1.00 (0.74–1.34)	0.70 (0.57–0.87)	0.91 (0.76–1.09)
Quintile 3	1.22 (0.97–1.52)	1.22 (0.93–1.59)	0.84 (0.69–1.01)	0.93 (0.78–1.10)
Quintile 4	1.19 (0.95–1.48)	1.10 (0.84–1.43)	0.77 (0.64–0.94)	0.95 (0.80–1.13)
Quintile 5 (highest)	1.66 (1.34–2.05)	1.08 (0.83–1.42)	0.70 (0.58–0.84)	0.89 (0.75–1.05)
Sex				
Women	1	1	1	1
Men	0.92 (0.82–1.03)	0.98 (0.85–1.14)	0.73 (0.65–0.82)	0.89 (0.81–0.97)
Other	0.95 (0.49–1.83)	1.58 (0.77–3.24)	1.98 (1.14–3.43)	1.17 (0.69–1.97)
Age (years)				
18–29	1	1	1	1
30–39	0.97 (0.81–1.17)	0.49 (0.40–0.60)	1.28 (1.03–1.60)	1.04 (0.88–1.21)
40–49	0.90 (0.75–1.08)	0.40 (0.32–0.49)	1.50 (1.21–1.86)	0.97 (0.83–1.13)
50–59	0.94 (0.78–1.13)	0.59 (0.48–0.72)	1.60 (1.29–1.98)	0.92 (0.78–1.08)
60–69	0.88 (0.71–1.09)	0.75 (0.59–0.94)	1.62 (1.28–2.04)	0.93 (0.78–1.11)
70 or more	0.73 (0.55–0.97)	0.57 (0.36–0.88)	1.43 (1.09–1.88)	0.91 (0.73–1.14)
Living situation				
Living alone	1	1	1	1
With partner/partner and children/adult family members	1.12 (0.98–1.27)	0.92 (0.78–1.09)	0.96 (0.86–1.08)	0.80 (0.72–0.88)
With children and without a partner	0.99 (0.76–1.28)	1.25 (0.93–1.70)	0.96 (0.74–1.23)	1.12 (0.91–1.37)
In a shared house with non‐family members/other	1.24 (1.01–1.53)	1.22 (0.95–1.56)	0.84 (0.67–1.05)	1.02 (0.86–1.21)
Born overseas (*v* born in Australia)	1.17 (1.05–1.31)	1.02 (0.88–1.18)	1.09 (0.98–1.22)	1.02 (0.93–1.12)
Main occupation (before COVID‐19)				
Paid employment (full or part time)	1	1	1	1
Unpaid work caring for children/dependent relatives only, or unemployed	0.67 (0.55–0.81)	NA	1.40 (1.20–1.64)	1.25 (1.09–1.44)
Student	1.05 (0.89–1.23)	1.56 (1.32–1.85)	0.96 (0.80–1.15)	1.42 (1.24–1.63)
Retired	0.70 (0.58–0.83)	0.11 (0.08–0.15)	1.27 (1.08–1.48)	1.05 (0.91–1.20)

NA = not applicable. Raw results: Supporting Information, table 1.

* Model included all four COVID‐19 experience types and state, remoteness and socio‐economic status quintile of residence, sex, age group, living situation, place of birth, and employment status.

† Index of Relative Socio‐economic Advantage and Disadvantage.

Respondents in major cities were more likely to have lost jobs because of the restrictions than people in rural or regional areas; respondents aged 18–29 years were more likely than older respondents, and students more likely than those in paid positions, to have lost jobs. ACT residents were less likely than those in NSW to have lost jobs (Box 3).

High worry about contracting COVID‐19 was most common among respondents in the lowest socio‐economic quintile; it was more common among people who were unemployed, doing unpaid work caring for children or dependent relatives, or retired than among those in paid employment; and among those who did not identify as male or female. Respondents aged 18–29 years were less frequently worried about contracting COVID‐19 than people in other age groups (Box 3).

Experiencing a high negative impact from COVID‐19 restrictions was more likely for respondents in major cities than those in regional or remote areas; for people living alone; for those who were students or unemployed or doing unpaid work caring for children or dependent relatives than for respondents in paid employment; and for women than men (Box 3).

### Mental health symptoms and optimism about the future

Clinically significant symptoms of depression were reported by 3791 respondents (estimated proportion, 27.6%; 95% CI, 26.1–29.1%) and mild symptoms by 3440 (26.5%; 95% CI, 25.1–27.8%). Clinically significant symptoms of generalised anxiety were reported by 3661 respondents (21.0%; 95% CI, 19.6–22.4%) and mild symptoms by 2774 (24.5%; 95% CI, 23.3–25.8%). A total of 1075 people (8.9%; 95% CI, 8.1–9.9%) reported having thoughts of being better off dead or self‐harm (PHQ‐9, item 9) on several days and 617 (5.7%) that they had such thoughts more frequently; 5277 (35.5%; 95% CI, 34.0–37.0%) reported increased irritability (GAD‐7, item 7) on several days, and 3058 (23.7%) more frequently. On the other hand, high optimism (score ≥ 8) was reported by 4075 respondents (28.3%; 95% CI, 27.1–29.6%) (Box 4).

Box 4 **Respondents’ self‐assessment of mental health symptoms during the past two weeks**

**Table:** 

Characteristic	Number	Estimated proportion (95% CI)
**Patient Health Questionnaire 9 (PHQ‐9)**		
Total score, mean (95% CI)	6.8 (6.6–7.0)	
Moderate/moderately severe/severe (clinically significant) symptoms (score, ≥ 10)	3791	27.6% (26.1–29.1%)
Mild symptoms of depression (score, 5–9)	3440	26.5% (25.1–27.8%)
Item 9: Thoughts of being better off dead or of self‐harm	1692	14.6% (13.5–16.0%)
Several days	1075	8.9% (8.1–9.9%)
More than half the days	356	3.0% (2.5–3.6%)
Nearly every day	261	2.7% (2.1–3.4%)
**Generalised Anxiety Disorder Scale (GAD‐7)**		
Total score, mean (95% CI)	5.5 (5.3–5.7)	
Moderate/ severe (clinically significant) symptoms (score, ≥ 10)	3661	21.0% (19.6–22.4%)
Mild anxiety symptoms (score, 5–9)	2774	24.5% (23.3–25.8%)
Item 7: Becoming easily annoyed or irritable	8335	59.2% (57.6–60.7%)
Several days	5277	35.5% (34.0–37.0%)
More than half the days	1925	14.6% (13.5–15.7%)
Nearly every day	1133	9.1% (8.1–10.3%)
**Optimism about future**		
Total score, mean (95% CI)	6.1 (6.0–6.2)	
High optimism (score, ≥ 8)	4075	28.3% (27.1–29.6%)

CI = confidence interval.

Weighted by state, socio‐economic status decile, sex, and age.

### Associations between COVID‐19 experiences and self‐reported mental health symptoms

After adjusting for state, remoteness, socio‐economic quintile of residence, sex, age group, living situation, place of birth, and employment status, people who had direct experience of COVID‐19 were more likely to report clinically significant anxiety than those who had not. Respondents who had lost jobs and people who were very worried about contracting COVID‐19 were more likely to report clinically significant symptoms of depression and anxiety, thoughts that they would be better off dead, and irritability. People for whom the restrictions had exerted a highly negative impact on daily life were more likely to report clinically significant signs of depression and anxiety, thoughts of self‐harm, and increased irritability. Optimism was more common among people without direct experience of COVID‐19, those who had not lost jobs, and people who did not find the COVID‐19 restrictions too difficult (Box 5).

Box 5 Associations between experience of COVID‐19 and COVID‐19 restrictions and mental health parameters in the past two weeks: adjusted odds ratios* with 95% confidence intervals

**Table:** 

Experience of COVID‐19 and restrictions	Mental health parameter				
	Clinically significant symptoms of depression	Clinically significant symptoms of anxiety	Thoughts of self‐harm or being better off dead	Easily annoyed or irritable	Great optimism about the future
Any experience	1.06 (0.95–1.19)	1.15 (1.02–1.30)	0.99 (0.86–1.14)	1.08 (0.98–1.20)	0.93 (0.84–1.03)
Job lost because of restrictions	1.50 (1.31–1.72)	1.22 (1.06–1.41)	1.31 (1.11–1.55)	1.22 (1.07–1.40)	0.76 (0.66–0.88)
Greatly worried about contracting COVID‐19	1.80 (1.61–2.00)	2.57 (2.30–2.87)	1.41 (1.23–1.61)	1.49 (1.34–1.65)	0.81 (0.72–0.90)
Great negative impact of restrictions	3.15 (2.88–3.44)	3.18 (2.89–3.49)	2.19 (1.96–2.45)	2.17 (1.98–2.37)	0.67 (0.61–0.74)

Raw results: Supporting Information, table 2.

* Model included all four COVID‐19 experience types and state, remoteness and socio‐economic status quintile of residence, sex, age group, living situation, place of birth, and employment status.

## Discussion

We have reported the first estimates of population levels of clinically significant symptoms of depression and anxiety among adults during the first month of COVID‐19 restrictions in Australia. These data suggest a widespread change in the mental health of the Australian adult population. About one‐quarter of respondents reported mild to moderate symptoms of depression or anxiety, which is substantially higher than found by a survey of American adults during 2005–2008 (subthreshold depression, 17%)^5^ or by a 2014 systematic review (median point prevalence of subthreshold anxiety, 4.4%).^6^ The point prevalence of clinically significant symptoms of depression (27.6%) was much higher than reported for a randomly selected Australian adult population (aged 32–58 years; 3.7%)^7^ and for other high income countries (3.3–10.8%; Supporting Information, table 3). Few estimates of the community prevalence of thoughts of being better off dead have been published; the 14.6% we found was much higher than the 1.8% for a random sample of South Australians in 2000.^8^

The mental health symptoms we assessed are indicators of normal psychological adjustment to abnormal circumstances that challenge the adaptive capacity of individuals, and reduce their access to social support and opportunities for participation. Depression and thoughts of being better off dead are most likely when people experience loss and feel trapped, humiliated, and powerless.^9^, ^10^, ^11^ “Disenfranchised grief” describes experiences of loss that might not be recognised by the individual affected or by others.^12^, ^13^ Everybody experienced some loss of liberty, autonomy, and agency as everyday activities were limited by the COVID‐19 restrictions; privacy was affected by the close scrutiny of adherence to prescribed health behaviours that, paradoxically, required isolation. Many people missed events of lifetime significance: weddings, end‐of‐life support for loved ones, milestone celebrations. Occupational identity and the ability to earn an income are fundamental to individuality, sense of purpose, and autonomy in adults; their loss is a profound one, leading to demoralisation and depression. Unrecognised losses that do not attract the social support or rituals that follow bereavement can induce powerlessness rather than the problem‐solving needed to manage psychological pain. Anxiety is increased by threat, danger, and uncertainty; the absence of definite knowledge about the evidence underlying specific restrictions and their duration contributed to uncertainty.

Our data indicate that some groups were especially vulnerable to mental health problems during the COVID‐19 restrictions: women and people aged 18–29 years; people living in regional and rural areas or in the lowest socio‐economic positions, and those not in paid employment before the pandemic; people who had lost jobs or opportunities for study; people living alone, who have fewer opportunities for daily interactions with family and friends; and people whose main occupation is to provide unpaid care for children or other dependent family members.

The consequences for occupational and social functioning are highly relevant to national recovery. People with these mental health problems are less motivated, energetic, socially engaged, and confident, and less able to concentrate, plan, organise, or trust. A public health approach has been essential to containing COVID‐19, and our findings indicate that a public mental health approach is needed for recovery.^14^ This would include universal interventions for the entire population, selective strategies for people with psychological problems, and indicated interventions for those with specific risks or needs.

As the mental health problems we report were associated significantly with the perceived risk of contracting COVID‐19 and the consequences of the restrictions, some improvement is expected as the pandemic ebbs and restrictions are lifted. However, universal, psychologically informed mental health strategies will still be needed. Strict messages and public policies ensured that distancing and isolation restrictions were observed; political and civic leaders acknowledging the magnitude and the psychological costs of individual contributions to the public good might now be helpful in reducing social suffering.^15^

Social relationships are predicated on trust, but when everyone is suspected of being able to transmit disease, trust in relationships is diminished. Activities providing engagement with other people offer essential opportunities for discussing life situations, experiencing empathy, and exploring solutions. Experiencing empathy is less likely during social media interactions than in personal encounters.^16^ Clear messages about safe social engagement with others are needed to reassure people, particularly those who are living alone or are afraid of contracting the disease. Government and non‐government agencies accompanied the restrictions with advice about the benefits for mental health of maintaining routines, social connections, and exercise, and the potential harms of isolation, lack of access to purposeful activities, and increased alcohol consumption.^17^ Similar guidance is needed about recapturing agency and resuming healthy social and economic participation, and the need for an adjustment period.^18^, ^19^

The mental health of people who have lost jobs will benefit from empathic, courteous, and encouraging assistance that does not rely exclusively on their own initiative for finding employment. Strengthening the psychological skills of staff and embedding mental health workers in employment agencies would be more effective than expecting people seeking work to also attend health services.

Our survey findings suggest that it would be appropriate for primary care clinicians to assess symptoms of depression and anxiety, and ideas of self‐harm, in people in the more vulnerable groups we have identified. Increased access to mental health care should be provided to people whose symptoms are not ameliorated by universal or selective mental health promotion strategies. Telehealth consultations should be recommended with caution; they require internet access, an appropriate personal device, and privacy, none of which are assured for people in lower socio‐economic positions. Integrating mental health care into community services can reduce barriers to access.

### Strengths and limitations

Our respondents comprised a large and diverse sample of people in Australia; we weighted their responses according to the characteristics of the national population, employed standardised psychometric measures that permit comparisons with other populations, and could distinguish worry about contracting COVID‐19 from the impacts of restrictions.

As participation in the survey was self‐selective, the representativeness of the sample cannot be assessed, nor a response rate calculated. As for all online surveys, it was less accessible to people with lower computer proficiency or English fluency, without internet access, or in lower socio‐economic positions; their experiences may have been underestimated. On the other hand, although the survey was advertised in neutral terms, people with mental health problems may have been more likely than others to complete it, leading to overestimation of symptom prevalence. A short, structured survey cannot gather nuanced information about mental health, and our data are not diagnostic; estimates of symptom prevalence based on self‐reports are generally higher than those based on clinical interviews.^20^ While thoughts of being better off dead were assessed with a single question, we did not assess suicide intent. Cross‐sectional surveys identify associations, not causal relationships. Nevertheless, our data provide an indication of the consequences of the first month of restrictions for the mental health of Australians and could inform public health planning and clinical service responses.

### Conclusion

The United Nations’ policy brief, *COVID‐19 and the need for action on mental health* (May 2020), concluded that the pandemic is leading to a “major mental health crisis”, and that mental health is a priority for which each country must urgently plan a response.^21^

## Competing interests

No relevant disclosures.

## Supporting information

Supplementary methods and results
