# Supplementary material for: Mental health of people in Australia in the first month of COVID‐19 restrictions: a national survey
Source: Med J Aust. 2020 Oct 26;213(10):458–64. doi: 10.5694/mja2.50831 (PMC7756394; doi:10.5694/mja2.50831)
Supplement: Supplementary file 1 — Supplementary methods and results [file MJA2-213-458-s001.pdf]

The Medical Journal of Australia

Supporting Information

Supplementary methods and results

This appendix was part of the submitted manuscript and has been peer reviewed.
It is posted as supplied by the authors.

Appendix to: Fisher JRW, Tran TD, Hammarberg K, et al. Mental health of people in Australia in the first month of COVID-19 restrictions: a national survey. *Med J Aust* 2020; doi: 10.5694/mja2.50831.

Sample size calculation 2
Data source 2
Procedure 3
Data management and analysis 3
Survey 5
Survey homepage in Monash University website 18
Advertisement in Facebook 20
Table 1. Characteristics of respondents with direct experiences of COVID-19: number........24
Table 2 Crosstabs between experience of COVID-19 and COVID-19 restrictions and mental health parameters in the past two weeks: number 26
Table 3 Summary of comparison data 21

Sample size calculation

A sample size of 8538 people was required to estimate the population prevalence rates in this study. The sample was calculated using the following formula.

$$Sample\ size = z^2 * \frac{p * (1 - p)}{e^2} * DEFF$$

Where:

- z (z-score) = 1.96 at a desired confidence level of 95%
- p = estimated prevalence. This study estimated many prevalence rates, so an estimated prevalence rate of 50% was used in this sample size calculation because it gives the largest possible sample size that satisfies all prevalence estimations.
- e = Margin of error at 1.5%

DEFF: design effect that is an adjustment made to calculate a sample size for a survey that the participants were selected using not a simple random method (in this study, respondent driven sampling). We selected a design effect of 2 in this study.

Data source

A questionnaire including study-specific, fixed response option questions and widely used standardised psychometric instruments.

Mental health outcomes

Psychological symptoms experienced over the previous fortnight were assessed using the Patient Health Questionnaire 9 (PHQ-9), the Generalised Anxiety Disorder Scale (GAD-7), and a study-specific questions assessing optimism about the future.

i. Patient Health Questionnaire 9 (PHQ-9)

The PHQ-9[1] is an easily understood self-report 9-item scale asking respondents to endorse each depressive symptom as “0” (not experienced) to “3” (experienced nearly every day). Aggregated responses yield a scale indicative of symptom severity. Formally validated against diagnostic psychiatric interviews, a PHQ-9 score ≥10 has sensitivity of 88% and specificity of 88% for Major Depression. PHQ-9 scores of 5-9 represent mild, 10-14 moderate, 15-19 moderately severe, and ≥20 severe depressive symptoms. PHQ-9 Item 9 asks whether the respondent has experienced ‘Thoughts that you would be better off dead or of hurting yourself in some way’.

ii. Generalised Anxiety Disorder Scale (GAD-7)

The GAD-7[2] is a 7-item scale assessing common symptoms of anxiety that uses same response options as PHQ-9 and is easily understood and acceptable. In formal validation against psychiatric interviews, a GAD-7 score ≥10 has sensitivity of 89% and specificity of 82% to detect Generalised Anxiety Disorder. Scores of 5-9 represent mild, 10-14 moderate, and 15-21 severe anxiety. Higher scores are strongly associated with functional impairment. GAD-7 Item 7 asks whether the respondent is ‘Becoming irritable or easily annoyed’.

iii. Optimism about the future

Optimism about the future was assessed by a visual analogue scale from 0 (not at all optimistic) to 10 (extremely optimistic).

Experience of COVID 19 and the COVID-19 restrictions

Study-specific questions assessed:

i. Direct experience of COVID-19: whether the respondent had been diagnosed with or tested for COVID-19, or lived with or knew someone with COVID-19: yes / no.
ii. Whether a job had been lost because of COVID-19 restrictions: yes / no.
iii. Worry about contracting COVID-19: a visual analogue scale with scores from 0 (not at all worried) to 10 (extremely worried).
iv. How badly COVID-19 restrictions had affected daily life: a visual analogue scale with scores from 0 (not at all badly) to 10 (very badly).

Socio-demographic characteristics

Study-specific questions with fixed response options were used to ascertain age, postcode, gender, whether born overseas or in Australia, living circumstances, and occupation.

Data on State, urban/rural residence, and Index of Relative Socio-economic Advantage and Disadvantage were derived from respondent's postcode using the most recent Australian Bureau of Statistics[3] data.

Procedure

The survey was built in Qualtrics Insight Platform. Titled How Are You? Living with COVID-19 Restrictions in Australia, it was available online from 3 April 2020, four days after stage two COVID-19 restrictions were implemented, to midnight on 2 May 2020. A link to the survey was hosted on the Monash University website (https://www.monash.edu/medicine/living-with-covid-19-restrictions-survey) and information about it was distributed on news[15,16] and social media and through organisational and personal networks, including the Facebook page of the Monash University Global and Women's Health Unit (GWH).

Data management and analysis

The outcomes were whether, in the last fortnight, the respondent had experienced:

1. Clinically significant symptoms of depression: PHQ-9 scores ≥ 10.
2. Clinically significant symptoms of anxiety: GAD-7 scores ≥ 10.
3. Any thoughts of being better off dead or self-harm: PHQ-9 item 9 score > 0
4. Becoming easily annoyed or irritable: GAD 7 item 6 score > 0
5. High optimism about the future: scores ≥ 8.

The visual analogue scales were each categorised into two groups: not at all or none to moderate (0-7) and high (≥ 8)

Data were analysed in three stages.

1. Population prevalence rates and 95% CIs of the outcomes, as well as the experiences of COVID-19 and consequent restrictions, were estimated, adjusting for differences in socio-demographic characteristics between the sample and the Australian population. The adjustment was made using weights for proportions of age groups, genders, Index of Relative Socio-economic Advantage and Disadvantage deciles, and states in the sample and the corresponding information in the population (Australian Bureau of Statistics, 2019).[3]

2. Characteristics of respondents with experiences of COVID-19 and the COVID-19 restrictions were examined using multiple logistic regressions. Reported direct experiences of COVID-19, lost jobs, being very worried about contracting COVID-19, and highly adverse impacts of the restrictions were dependent variables and socio-demographic characteristics were the independent variables of the models.

3. Multiple logistic regression analyses were performed to examine associations between each of the mental health outcomes (dependent variables) and experiences of COVID-19 and the COVID-19 restrictions (independent variables), taking into account socio-demographic characteristics (independent variables).

Only complete data were included in analyses, which were conducted using STATA Version 16 (StataCorp). Comparisons were made with nationally representative population data generated with the same instruments with adults in Australia and other high-income nations (Table 1).

Approval to conduct the study was provided by [Institution] University Human Research Ethics Committee (2020-24080-42716)

Survey

MONASH University

HOW ARE YOU?

Living With COVID-19 Restrictions in Australia

(Monash University Human Research Ethics project ID: 24080)

***

To help our governments and the community to understand what life is like during the COVID-19 restrictions, we want as many people as possible to complete this snapshot survey.

This survey is anonymous. We can't know who you are.

We would like to hear from you if you are **18 or older** and **live in Australia**!

It will take only about 10 minutes to answer the questions.

If you want to find out more about the survey before you begin, please click here for more information.

To begin the survey, please click "NEXT PAGE".

Next Page

Are you living in Australia?

- ○ Yes
- ○ No

Previous Page | Next Page

Are you above 17 years of age?

- ○ Yes
- ○ No

Previous Page | Next Page

The first group of questions asks about you and your situation

1. How old are you? (years)

2. Do you live:

- ◯ On your own
- ◯ With only your partner
- ◯ With your partner and children
- ◯ With children and without a partner
- ◯ With adult family members
- ◯ In a shared house with non-family members
- ◯ Other

Previous Page

Next Page

3. What is your residential postcode?

4. Are you:

- ○ Female
- ○ Male
- ○ Other

5. Were you born in Australia?

- ○ Yes
- ○ No

Previous Page

Next Page

The next set of questions asks about your experience of COVID-19 pandemic

6. To what extent have you experienced COVID-19?

(Please answer all items in this question)

	Yes	No
I have been treated in hospital for COVID-19	○	○
I have had COVID-19 but did not have to go to hospital	○	○
I have been tested for COVID-19	○	○
Someone who lives with me has or has had COVID-19	○	○
Someone I know who doesn't live with me has or has had COVID-19	○	○

Previous Page | Next Page

7. How worried are you that you will catch COVID-19?

Not at all worried — Extremely worried

0	1	2	3	4	5	6	7	8	9	10
○	○	○	○	○	○	○	○	○	○	○

Previous Page | Next Page

8. What is your situation at the moment?

(Please answer all items in this question)

	Yes	No
I have a job and am working from home	○	○
I have a job that I need to leave home to do	○	○
I am doing unpaid work caring for children	○	○
I am doing unpaid work caring for dependent relatives	○	○
I have lost my job because of COVID-19	○	○
I was unemployed before COVID-19	○	○
I am retired	○	○
I am a student and my course is delivered online	○	○
I am a student but my course has been suspended	○	○
My main source of income is government benefits	○	○

Previous Page Next Page

9. How badly have the COVID-19 restrictions affected your daily life?

Not at all										Very badly
0	1	2	3	4	5	6	7	8	9	10
○	○	○	○	○	○	○	○	○	○	○

10. Since COVID-19 I am drinking alcohol:

- ○ More than I used to
- ○ Less than I used to
- ○ About the same
- ○ I don't drink alcohol

The next set of questions is about your health in general

11. Have you been able to get the care you need for non-COVID-19 health conditions or a disability?

- ○ Yes; there's been no change in my health or disability care
- ○ Yes; my health or disability care has been better
- ○ No; my health or disability care has been worse
- ○ I haven't needed health or disability care

Previous Page Next Page

The next set of questions is about how you have felt in the last two weeks

12. Over the last 2 weeks, how often have you been bothered by any of the following problems?

(Please answer all items in this question)

	Not at all	Several days	More than half the days	Nearly every day
a. Little interest or pleasure in doing things	O	O	O	O
b. Feeling down, depressed, or hopeless	O	O	O	O
c. Trouble falling or staying asleep, or sleeping too much	O	O	O	O
d. Feeling tired or having little energy	O	O	O	O
e. Poor appetite or overeating	O	O	O	O
f. Feeling bad about yourself, or that you are a failure, or have let yourself or your family down	O	O	O	O
g. Trouble concentrating on things, such as reading the newspaper or watching television	O	O	O	O
h. Moving or speaking so slowly that other people could have noticed. Or the opposite, being so fidgety or restless that you have been moving around a lot more than usual	O	O	O	O
i. Thoughts that you would be better off dead, or of hurting yourself in some way	O	O	O	O

Previous Page

Next Page

13. Over the last 2 weeks, how often have you been bothered by any of the following problems?

(Please answer all items in this question)

	Not at all	Several days	More than half the days	Nearly every day
a. Feeling nervous, anxious or on edge	○	○	○	○
b. Not being able to stop or control worrying	○	○	○	○
c. Worrying too much about different things	○	○	○	○
d. Trouble relaxing	○	○	○	○
e. Being so restless that it is hard to sit still	○	○	○	○
f. Becoming easily annoyed or irritable	○	○	○	○
g. Feeling afraid as if something awful might happen	○	○	○	○

Previous Page   Next Page

The last set of questions is about the impact of COVID-19 on your life:

14. Please tell us up to three bad things that have happened to you because of the COVID-19 restrictions

Bad thing 1:

Bad thing 2:

Bad thing 3:

Previous Page

Next Page

15. Please tell us up to three good things that have happened to you because of the COVID-19 restrictions

Good thing 1:

Good thing 2:

Good thing 3:

Previous Page

Next Page

16. Please tell us in general how optimistic you feel about the future

Not at all optimistic										Extremely optimistic
0	1	2	3	4	5	6	7	8	9	10
○	○	○	○	○	○	○	○	○	○	○

17. Please write anything else you would like us to know about your experience of COVID-19 (up to 250 characters)

Characters remaining: 250

Please click "NEXT PAGE" to submit the survey.

Previous Page

Next Page

Thank you for completing the survey. Your response has been recorded. Please encourage other people to complete it by sending this link: https://tinyurl.com/Covid19-Restrictions

We will give the results to governments and other organisations to help them understand what people need now and to prepare for similar circumstances in the future.

You can see the survey results in a few weeks here.

If you are feeling distressed, there are places you can contact for help:

Your GP
Beyond blue (phone number: 1300 22 4636)
Lifeline (phone number: 13 11 14)
For advice and information, go to: Government of Australia

Survey homepage in Monash University website

MONASH University

LIVING WITH COVID-19 RESTRICTIONS IN AUSTRALIA

Home | How are you? | About the Survey | Research and funding | Recognising disenfranchised grief amid COVID-19 | Contact us

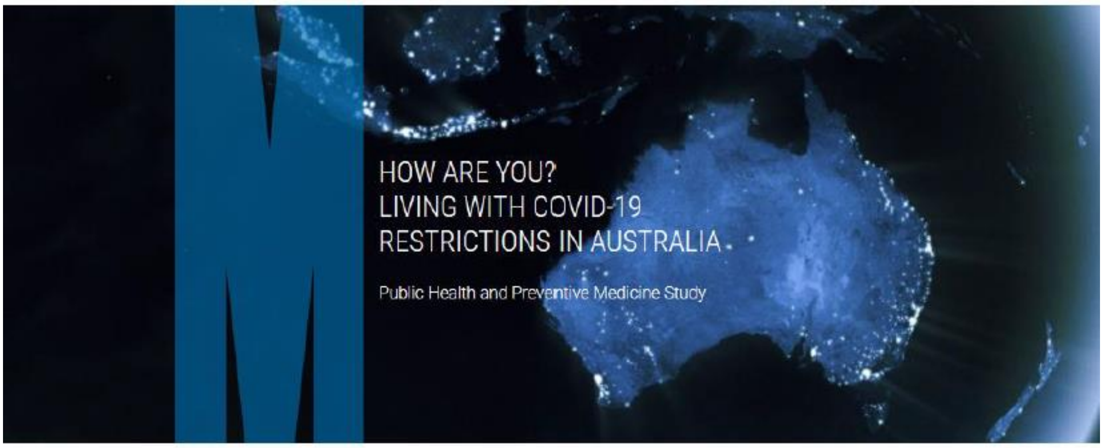

HOW ARE YOU? LIVING WITH COVID-19 RESTRICTIONS IN AUSTRALIA

Monash University Human Research Ethics project ID: 24080

Australia has never experienced anything on the scale of COVID-19 and the temporary restrictions designed to limit the spread. These are likely to affect everyone's sense of wellbeing.

Monash University's School of Public Health and Preventive Medicine has created a short, anonymous, online survey to measure the ways in which COVID-19 is affecting people.

If you are over 18 and living in Australia, we want to know about your experiences with COVID-19 restrictions.

Please complete the survey here.

ABOUT THE SURVEY

The survey asks questions about your life during the COVID-19 crisis. The survey is anonymous. We can't know who you are.

YOU NEED TO BE AT LEAST 18 YEARS OLD and LIVING IN AUSTRALIA to participate in the survey.

The survey takes about 10 minutes to complete.

There will be a second survey a few weeks later. The third survey will take place after all the restrictions are lifted.

We encourage you to answer the questions every time they're posted. However, it's still helpful if you do it only once or twice.

The survey will be available online for about two weeks each time.

The results of the survey will be made available on this website. Be sure to visit this site again to check for the results.

RESEARCH AND FUNDING

This study is being led by Professor Jane Fisher, Director, and Dr Maggie Kirkman, Senior Research Fellow, from the Global and Women's Health unit at Monash University's School of Public Health and Preventive Medicine.

The research will help us to understand what it's like to live with the temporary limits on normal life to protect us all from COVID-19. We will share the results widely to help governments and other organisations understand what people need now and to prepare for the future.

Alongside questions about potential negative impacts, we ask about any benefits you've experienced from the COVID-19 restrictions.

This research was made possible by a generous donation from Professor John McBain and Dr Penny Foster. Neither the researchers nor the donors have any conflict of interest in the research.

RECOGNISING DISENFRANCHISED GRIEF AMID COVID-19

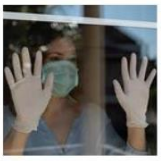

Professor Jane Fisher and Dr Maggie Kirkman recently applied their knowledge to the coronavirus pandemic, sharing their thoughts on the problems associated with disenfranchised grief in Monash's online publication Lens.

Disenfranchised grief is the term applied to experiences of loss that might not be recognised, either by the person experiencing the loss or by others. As with recognised grief (such as the death of a loved one), disenfranchised grief is accompanied by disbelief and shock, wishing reality was different or as it was before the loss, and then uncertainty and sadness as reality grows. Disenfranchised grief can be more psychologically damaging than grief that is recognised and supported.

More information

There are places you can contact for help and information:

- Your GP
- Beyond Blue: beyondblue.org.au
- Government of Australia: australia.gov.au

CONTACT US

School of Public Health and Preventive Medicine, Monash University

If you would like to know more about the survey, you can contact the researchers:

Professor Jane Fisher
Email: Jane.Fisher@monash.edu

Dr Maggie Kirkman
Email: Maggie.Kirkman@monash.edu

Please complete the survey here.

MONASH University

Advertisement in Facebook

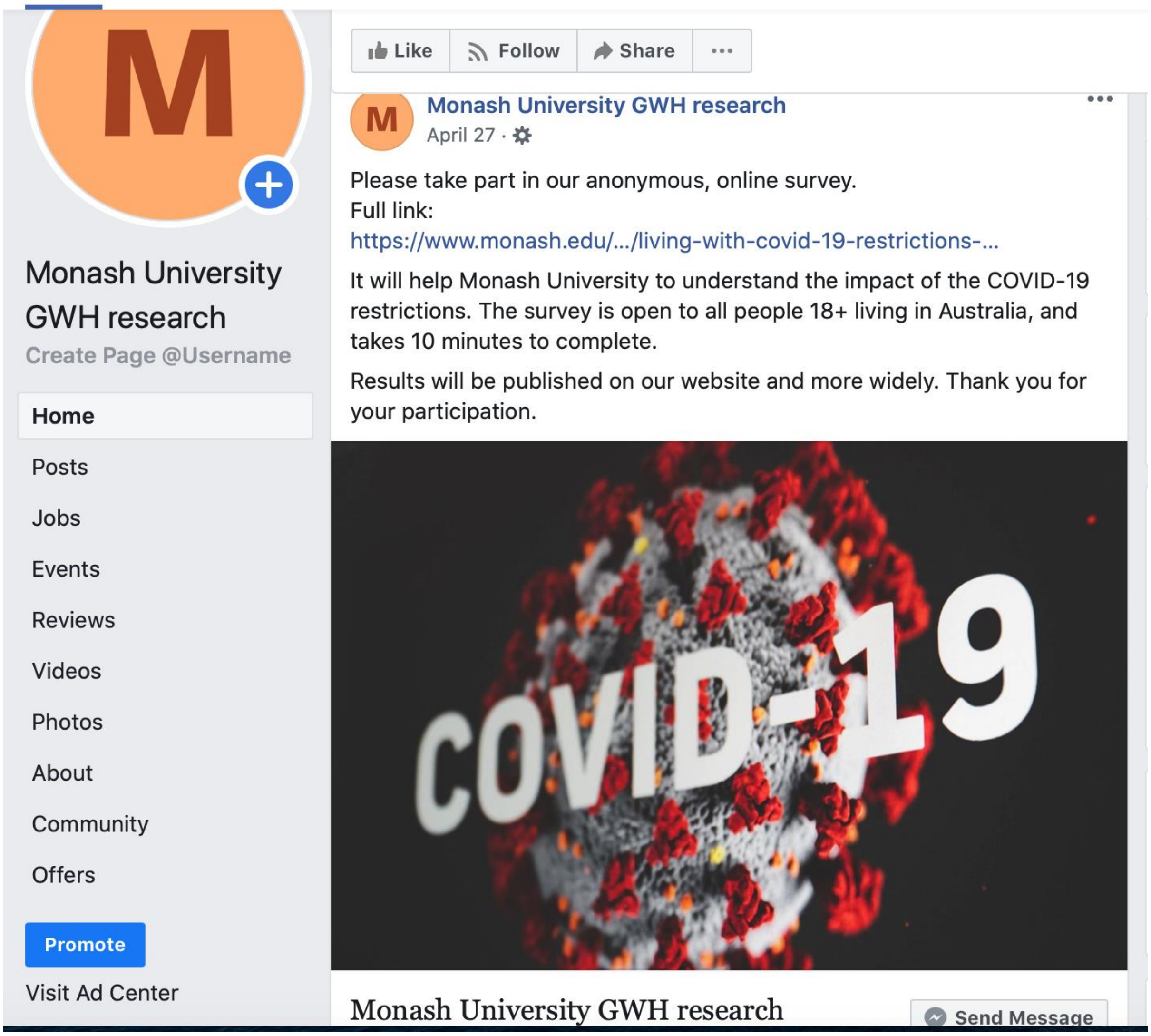

Table 1. Characteristics of respondents with direct experiences of COVID-19: number

	Any direct experience of COVID-19		Lost a job because of COVID-19		Greatly worried about contracting COVID-19		High negative impact of COVID-19 restrictions	
	**No**	**Yes**	**No**	**Yes**	**No**	**Yes**	**No**	**Yes**
State								
New South Wales	2258	495	2518	235	2296	457	2094	659
Victoria	5159	946	5505	600	5113	992	4445	1660
Queensland	1651	288	1752	187	1623	316	1453	486
Western Australia	1037	140	1069	108	1017	160	930	247
South Australia	704	132	777	59	703	133	655	181
Tasmania	380	65	410	35	384	61	349	96
Australian Capital Territory	399	66	445	20	410	55	375	90
Northern Territory	94	15	102	7	98	11	93	16
Major city or Regional/remote areas								
Major city	4139	645	4356	428	3998	786	3744	1040
regional/remote areas	7543	1502	8222	823	7646	1399	6650	2395
Socio-economic position†								
Quintile 1 (lowest)	963	130	1006	87	877	216	826	267
Quintile 2	1350	191	1418	123	1311	230	1197	344
Quintile 3	1918	310	2007	221	1845	383	1702	526
Quintile 4	2612	426	2749	289	2554	484	2244	794
Quintile 5 (highest)	4839	1090	5398	531	5057	872	4425	1504
Sex								
Women	8773	1661	9470	964	8704	1730	7773	2661
Men	2853	475	3051	277	2891	437	2575	753
Other	56	11	57	10	49	18	46	21
Age (years)								

	Any direct experience of COVID-19		Lost a job because of COVID-19		Greatly worried about contracting COVID-19		High negative impact of COVID-19 restrictions	
	**No**	**Yes**	**No**	**Yes**	**No**	**Yes**	**No**	**Yes**
18–29	1087	250	1059	278	1195	142	951	386
30–39	1896	398	2080	214	1977	317	1679	615
40–49	2385	469	2631	223	2404	450	2141	713
50–59	2564	500	2757	307	2550	514	2344	720
60–69	2451	382	2636	197	2319	514	2168	665
70 or more	1299	148	1415	32	1199	248	1111	336
Living situation								
Living alone	2302	358	2452	208	2203	457	1938	722
With partner/partner and children/adult family members	8105	1525	8798	832	8115	1515	7381	2249
With children and without a partner	492	86	510	68	485	93	403	175
In a shared house with non-family members/other	783	178	818	143	841	120	672	289
Born overseas or Born in Australia								
Born overseas	9075	1604	9708	971	9015	1664	8035	2644
Born in Australia	2607	543	2870	280	2629	521	2359	791
Main occupation (before COVID-19)								
Paid employment (full or part time)	6894	1436	7393	937	7102	1228	6341	1989
Unpaid work caring for children/dependent relatives only, or unemployed	1012	134	1146	0	918	228	827	319
Student	1096	247	1078	265	1172	171	910	433
Retired	2680	330	2961	49	2452	558	2316	694

Table 2. Crosstabs between experience of COVID-19 and COVID-19 restrictions and mental health parameters in the past two weeks: number

**Experience of COVID-19 and restrictions**	**Mental health parameter**									
	**Clinically significant symptoms of depression**		**Clinically significant symptoms of anxiety**		**Thoughts of self-harm or being better off dead**		**Easily annoyed or irritable**		**Great optimism about the future**	
	**No**	**Yes**	**No**	**Yes**	**No**	**Yes**	**No**	**Yes**	**No**	**Yes**
Any experience										
No	8830	2852	8830	2852	10261	1421	4727	6955	7838	3487
Yes	1559	588	1559	588	1876	271	767	1380	1497	588
Job lost because of restrictions										
No	9638	2940	9638	2940	11111	1467	5123	7455	8402	3799
Yes	751	500	751	500	1026	225	371	880	933	276
Greatly worried about contracting COVID-19										
No	9003	2641	9003	2641	10317	1327	4810	6834	7780	3531
Yes	1386	799	1386	799	1820	365	684	1501	1555	544
Great negative impact of restrictions										
No	8474	1920	8474	1920	9405	989	4603	5791	6770	3299
Yes	1915	1520	1915	1520	2732	703	891	2544	2565	776

Table 3 Summary of comparison data

Proportions of people scoring ≥ 10 (indicating point prevalence of moderate to severe depressive symptoms) and 4 – 9 (point prevalence mild to moderate symptoms) on PHQ-9 in the previous 30 days				
**Author/year**	**Country**	**Participants**	**≥ 10**	**4 - 9**
**Participants randomly selected from the general community**				
Patten & Schopflocher (2009)[4]	Canada	3304 people aged at least 18 years	3.3%	
Shim et al. (2011)[5]	USA	10,283 people aged at least 18 years	6.9%	16.97%
Johansson et al. (2013)[6]	Sweden	1329 people aged at least 18 years	10.8%	
Kocalevent et al. (2013)[7]	Germany	5018 people aged at least 18 years	5.6%	
Kiely & Butterworth (2015)[8]	Australia	546 people aged 32 to 35 years and 1515 aged 52 to 58 years	3%*	
**Participants selected from people attending general practices**				
Pirkis et al. (2009)[9]	Australia	>22,000 people aged >59 consulting a GP	8.2%	
Carey et al. (2014)[10]	Australia	1004 people consulting a GP	13%	
**Specific population groups**				
Farrer et al. (2016)[11]	Australia	611 university students	7.9%	

**Proportion of people scoring ≥ 10 on the GAD-7, indicating point prevalence of moderate to severe anxiety symptoms in the prior 30 days**				
**Author/year**	**Country**	**Participants**	**Point prevalence**	
**Participants randomly selected from the general community**				
Johansson et al. (2013)[6]	Sweden	1329 people aged at least 18 years	14.7% ≥8	
Hinz et al (2017)[12]	Germany	9721 people aged at least 18 years	5.9% ≥ 10	
**Specific population groups**				
Farrer et al. (2016)[11]	Australia	611 university students	17.5% ≥10	
Hammarberg et al (2019)[13]	Australia	4947 women aged > 49 years from the general community responding to an online survey	10.6% ≥10	
**Proportion of people reporting thoughts of wanting to die, or of contemplating self-harm 'recently'**				
**Author/year**	**Country**	**Participants**	**Point prevalence**	
**Participants randomly selected from the general community**				
Goldney et al (2000)[14]	Australia	2501 people in South Australia aged at least 18 years	1.8%	

PHQ-9: Patient Health Questionnaire 9
GAD-7: Generalised Anxiety Disorder Scale
GP: general practitioner
*Diagnosis of any depressive episode in prior 30 days detected with a sensitivity of 0.64 and a specificity of 0.92 by a PHQ 9 score ≥ 10

References

1. Kroenke K, Spitzer RL, Williams JBW. The PHQ-9: Validity of a brief depression severity measure. *J Gen Intern Med* 2001; 16: 606-13.
2. Spitzer RL, Kroenke K, Williams JBW, et al. A Brief Measure for Assessing Generalized Anxiety Disorder: The GAD-7. *Arch Intern Med* 2006; 166: 1092-7.
3. Australian Bureau of Statistics. 3101.0 - Australian Demographic Statistics, Sep 2019. Canberra: Australian Bureau of Statistics; 2019. https://www.abs.gov.au/AUSSTATS/abs@.nsf/DetailsPage/3101.0Sep%202019?OpenDocument (accessed May 2020).
4. Patten SB, Schopflocher D. Longitudinal epidemiology of major depression as assessed by the Brief Patient Health Questionnaire (PHQ-9). *Compr Psychiatry* 2009; 50: 26-33.
5. Shim RS, Baltrus P, Ye J, et al. Prevalence, treatment, and control of depressive symptoms in the United States: results from the National Health and Nutrition Examination Survey (NHANES), 2005-2008. *J Am Board Fam Med* 2011; 24: 33-8.
6. Johansson R, Carlbring P, Heedman as, et al. Depression, anxiety and their comorbidity in the Swedish general population: point prevalence and the effect on health-related quality of life. *PeerJ* 2013; 1: e98.
7. Kocalevent R-D, Hinz A, Brähler E. Standardization of the depression screener Patient Health Questionnaire (PHQ-9) in the general population. *Gen Hosp Psychiatry* 2013; 35: 551-5.
8. Kiely KM, Butterworth P. Validation of four measures of mental health against depression and generalized anxiety in a community based sample. *Psychiatry Res* 2015; 225: 291-8.
9. Pirkis J, Pfaff J, Williamson M, et al. The community prevalence of depression in older Australians. *J Affect Disord* 2009; 115: 54-61.
10. Carey M, Jones KA, Yoong SL, et al. Comparison of a single self-assessment item with the PHQ-9 for detecting depression in general practice. *Fam Pract* 2014; 31: 483-9.
11. Farrer LM, Gulliver A, Bennett K, et al. Demographic and psychosocial predictors of major depression and generalised anxiety disorder in Australian university students. *BMC Psychiatry* 2016; 16: 241.
12. Hinz A, Klein AM, Brähler E, et al. Psychometric evaluation of the Generalized Anxiety Disorder Screener GAD-7, based on a large German general population sample. *J Affect Disord* 2017; 210: 338-44.
13. Hammarberg K, Holton S, Michelmore J, et al. Thriving in older age: A national survey of women in Australia. *Maturitas* 2019; 122: 60-5.
14. Goldney RD, Wilson D, Grande ED, et al. Suicidal Ideation in a Random Community Sample: Attributable Risk Due to Depression and Psychosocial and Traumatic Events. *Aust N Z J Psychiatry* 2000; 34: 98-106.
15. Fisher J. How are you? Living with COVID-19 restrictions in Australia. *Lens* (Monash University, Melbourne) [online], 8 Apr 2020. https://lens.monash.edu/@medicine-health/2020/04/08/1380027/how-are-you-living-with-covid-19-restrictions-in-australia
16. Scott S, Kinsella E. Mental health and COVID-19: how the coronavirus is affecting our way of life. ABC Online, 18 Apr 2020. https://www.abc.net.au/news/2020-04-18/mental-health-and-coronavirus-how-australia-is-reacting-covid19/12159750
